# Revisiting the infectivity and pathogenicity of *Cryptosporidium avium* provides new information on parasitic sites within the host

**DOI:** 10.1186/s13071-018-3088-x

**Published:** 2018-09-19

**Authors:** Zhaohui Cui, Dan Song, Meng Qi, Sumei Zhang, Rongjun Wang, Fuchun Jian, Changshen Ning, Longxian Zhang

**Affiliations:** 1 College of Animal Science and Veterinary Medicine, Henan Agricultural University, Zhengzhou, 450002 China; 2 International Joint Research Laboratory for Zoonotic Diseases of Henan, Zhengzhou, China

**Keywords:** *Cryptosporidium avium*, Parasitic site, Bursa fabricii, Biology

## Abstract

**Background:**

*Cryptosporidium* spp. are protozoans that cause diarrheal illness in humans and animals, including birds, worldwide. The present study was aimed to revisit the infectivity and pathogenicity of *C. avium*, recently considered to be a valid avian-infecting species of *Cryptosporidium*, and foster further understanding of its biological characteristics.

**Results:**

Results showed that no *Cryptosporidium* oocysts were detected in the feces of experimentally inoculated BALB/c mice, Mongolian gerbils, quail or budgerigars within 30 days post-infection (dpi). Oocysts were first detected in feces of 3-day-old and 40-day-old hens at 8 and 9 dpi, respectively. In ducks infected with *C. avium*, oocysts were first detected at 9 dpi. Oocysts of infected animals were studied using a nested-polymerase chain reaction (PCR) technique for the *SSU* rRNA gene, actin gene, *HSP70* gene and *Cryptosporidium* oocyst wall protein gene (*COWP*) detection. Restriction fragment length polymorphism (RFLP), using *Ssp*I and *Vsp*I restriction enzymes, was carried out to genotype the species and obtained amplification products were sequenced. *Cryptosporidium* developmental stages were found in the longitudinal plica of the bursa fabricii (BF) of hens, with high levels observed in histological sections and scanning electron microscopy. No pathological changes were observed.

**Conclusions:**

These findings indicate that the bursa fabricii may be the primary site of *C. avium* infection. More biological data are needed to support the establishment of new species and contribute to the taxonomy of *Cryptosporidium.*

**Electronic supplementary material:**

The online version of this article (10.1186/s13071-018-3088-x) contains supplementary material, which is available to authorized users.

## Background

Cryptosporidiosis is one of the most common protozoal diseases of birds worldwide [1]. Four avian-adapted species of *Cryptosporidium* have been recognized in birds, including *Cryptosporidium baileyi*, *Cryptosporidium galli*, *Cryptosporidium meleagridis* and *Cryptosporidium avium* [2–5]. Additionally, cases of *Cryptosporidium hominis*, *Cryptosporidium parvum*, *Cryptosporidium muris* and *Cryptosporidium andersoni* have also been reported in birds [4, 6, 7]. Likewise, some genetically distinct avian genotypes have been identified in previous studies, including avian genotypes (I-IV, VI), goose genotypes (I-IV), a duck genotype, and a Eurasian woodcock genotype [6–14].

Cryptosporidiosis in birds has a wide spectrum of clinical signs, varying from asymptomatic to serious infection to death, and has been mainly associated with high morbidity and mortality in poultry [15, 16]. *Cryptosporidium baileyi* is the most commonly-reported species in birds, with clinical signs including dyspnea, coughing, sneezing and depression [17]. Infection with C*. galli* primarily causes diarrhea, chronic apathy, weight loss and high mortality [4]. However, in some birds infected with *C. galli*, no clinical signs were observed [18]. *Cryptosporidium meleagridis*, originally described in birds, is the only C*ryptosporidium* species reported in both natural and experimental infections in avian and mammalian species, as well as humans [19].

*Cryptosporidium avium*, previously known as *Cryptosporidium* avian genotype V, was recognized as a valid species in 2016 [5]. The morphology, biology and host specificity have been studied. As the classification of species within the genus *Cryptosporidium* is constantly updated, more data was needed to support the establishment of a new separate species of the genus *Cryptosporidium*. In the present study, we have revisited the infectivity and pathogenicity of *C. avium* using more animal species. Additionally, a new parasitic site has been discovered.

## Methods

### Source of oocysts

*Cryptosporidium* oocysts were obtained from the feces of naturally infected cockatiels (*Nymphicus holandicus*) in a pet market in the Province of Henan, China. Oocysts from cockatiels were pooled and used to infect 30 one-day-old Roman chickens. Oocysts from the 30 one-day-old Roman chickens were then used to infect other animals, after concentration using a water ether technique [20] and purification with discontinuous sucrose density centrifugation [21]. Oocysts were counted with a Neubauer hemocytometer. A combination of streptomycin and penicillin was added and this oocyst suspension was kept at 4 °C.

Morphometry analyses of *C. avium* oocysts were performed using digital analysis of images (Motic Images Plus 2.0 software). A 20 μl aliquot containing 10^5^ purified oocysts was examined for each measurement. The length and width of oocysts (*n* = 100) were measured under bright-field microscopy at 1000× magnification; these values were used to calculate the length-to-width ratio of each oocyst.

### DNA extraction and molecular analyses

Genomic DNA was extracted using an E.Z.N.A.® Stool DNA Kit (Omega Bio-tek, Norcross, GA, USA). Extracted DNA was stored at -20 °C until used in nested-polymerase chain reaction (PCR) assays. Nested-PCR protocols were used to amplify partial sequences of the *Cryptosporidium* small-subunit rRNA gene (*SSU*), actin gene, *HSP70* gene and *Cryptosporidium* oocyst wall protein gene (*COWP*), according to previous studies [22–25]. Negative (molecular grade water) and positive controls (DNA from *C. baileyi*) were included in each PCR amplification. *Cryptosporidium* species were also determined by PCR restriction fragment length polymorphism (RFLP) analysis, using *Ssp*I and *Vsp*I [8, 26]. The PCR products were detected by agarose gel (1.5%) electrophoresis, purified with GenElute™ Gel Extraction Kit (Sigma Aldrich, St. Louis, MO, USA) and sequenced in both directions with secondary primers using a Big Dye Terminator v.3.1 cycle sequencing kit and ABI Prism 3130 genetic analyzer (Applied Biosystems, Carlsbad, CA, USA). The sequences were assembled using the ChromasPro 2.64 (http://www.technelysium.com.au), and genotyped with a multiple-sequence alignment analysis together with reference sequences retrieved from the GenBank database using ClustalX 2.1 (http://www.clustal.org/).

### Experimental design

#### Animals

Thirty three-day-old and six 40-day-old Roman chickens, 30 ten-day-old quail (*Coturnix coturnix japonica*), 15 three-day-old Cherry Valley ducks, eight four-week-old BALB/c mice, eight four-week-old Mongolian gerbils (*Meriones unguiculatus*), and six 20-day-old budgerigars (*Melopsittacus undulatus*) were used for experimental infection studies. In addition, the same number of animals from each host species/strain was used as a negative control. All animals used in this study were obtained from the Henan Experimental Animal Center.

Each animal was confirmed to be free of *C. avium* infection by microscopic examination of feces. Animals were randomly divided into control and test groups, housed individually in plastic cages or bird cages under pathogen-free conditions, and received sterilized food and water. Each animal in the test group was inoculated orally by stomach tube with a dose of 1 × 10^6^ oocysts suspended in 500 μl of distilled water. Each animal used as negative control was inoculated with equal doses of distilled water. One *C. avium* positive animal from the three-day-old chicken group was euthanized 15 days post-infection (dpi). Tissue samples were processed for histology and scanning electron microscopy. The light and electron microscopic examination were performed according to a previous study with a slight modification [27].

#### Clinical status

Each animal in each group was examined daily for the appearance of clinical signs. Rectal temperature, breath, appetite and presence of any abnormal behaviors were observed.

#### Oocyst excretion

Fecal samples were obtained daily during the experiment, starting from the second dpi, to determine the prepatent period. The experiments were terminated at 30 dpi. The number of oocysts in each animal of all experimentally infected groups was counted by hemocytometer slide under bright-field microscopy at 400× magnification. Dynamics of oocyst shedding were determined as a number of oocysts per gram (OPG). The OPG was estimated on the basis of the number of oocysts counted [28].

### Histological examination

After a complete examination of all gastrointestinal organs at necropsy, organs and tissues collected from liver, trachea, stomach, duodenum, jejunum, ileum, cecum, colon and bursa fabricii (BF) were fixed in 10% buffered formalin for 24 h, dehydrated in absolute ethanol, cleared in xylene, and embedded in paraffin. Each tissue section was cut at a thickness of 4 μm, stained with hematoxylin and eosin (H&E), and observed microscopically at 1000× magnification by light microscopy.

### Scanning electron microscopy

To further observe and confirm *C. avium* colonization in chickens, tissue samples from the BF were selected for scanning electron microscopy (SEM) observation according to the results of histological observation. Samples were fixed in 2.5% glutaraldehyde for one week at 4 °C, and then washed with 0.1 mol/l phosphoric acid buffer (pH = 7.4) three times for 10 min each. The dehydration procedure followed conventional methods in a graded ethanol series of 30%, 50%, 70%, 90% and 100%, and two more changes of 100%, each for 5 min followed by 50% isoamyl acetate solution (v/v, isoamyl acetate: ethanol = 1:1) and 100% isoamyl acetate solution for 10 min, respectively. After specimens were critically point-dried using CO_2_ and coated with gold, observations were made using an S-3400 SEM (Hitachi, Tokyo, Japan).

## Results

### Clinical status

The appetites and attitudes of all animals in both experimental and control groups were normal during the experiment. All animals remained free of clinical signs at any point. No remarkable changes were observed in macroscopic observations. In addition, no animals died during the experiment.

### Oocyst morphology

**Fig. 1:**
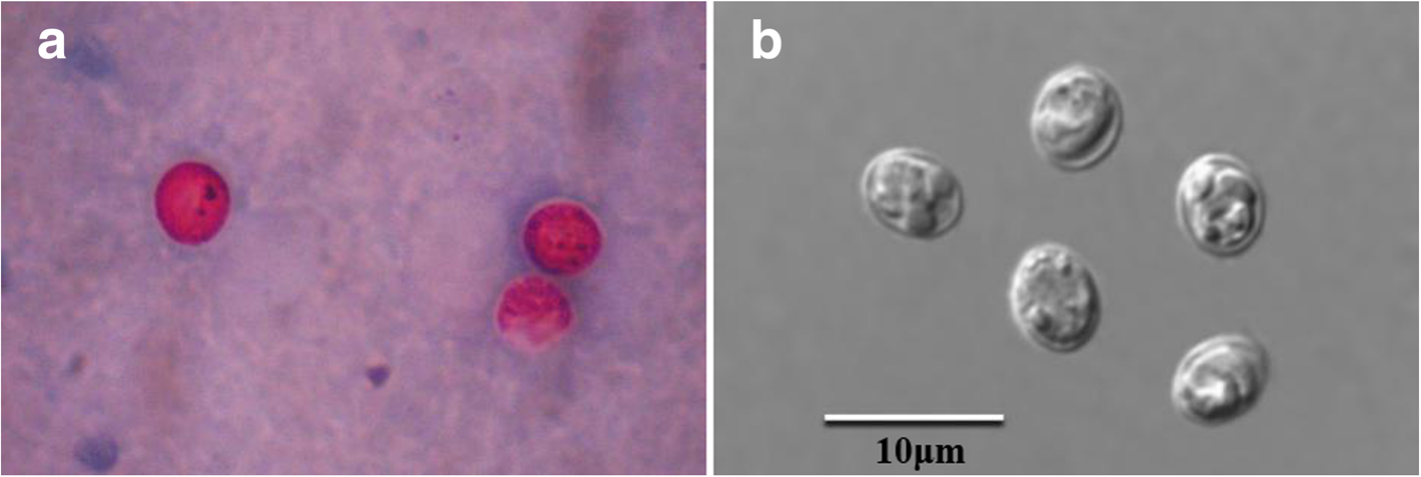
Oocysts in fecal smears showed typical *Cryptosporidium* characteristics when stained with Modified Ziehl-Neelsen stain (**a**, ×1000). Oocyst morphology was also observed by differential interference contrast (DIC) microscope (**b**, ×1000)

Oocysts of *C. avium* originated from naturally-infected red-crowned parakeets were morphometrically identical to those recovered from experimentally-infected hens, measuring 4.58–5.89 × 3.98–4.83 μm (mean 5.42 × 4.46 μm) with a length to width ratio of 1.22 (*n* = 100). Oocysts in fecal smears showed typical *Cryptosporidium* characteristics when stained with a Modified Ziehl-Neelsen stain (Fig. 1a). Moreover, oocyst morphology was observed by differential interference contrast (DIC) microscopy (Fig. 1b).

### Oocyst shedding

**Fig. 2:**
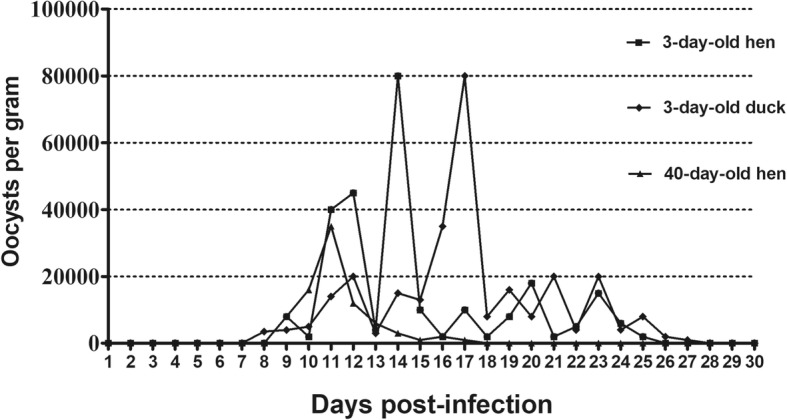
Excretion of oocysts in 1 g of feces in 3-day-old chickens, 3-day-old ducks and 40-day-old chickens infected with *C. avium* during the experiment (mean of all the examined animals)

No *Cryptosporidium* oocysts were detected in the feces of experimentally inoculated BALB/c mice, Mongolian gerbils, quail or budgerigars within 30 dpi. However, fecal examination of chickens and ducks revealed fully sporulated *C. avium* oocysts. Oocysts were first detected in the feces of 3-day-old chickens at 8 dpi, peaking twice at 11 and 14 dpi. The infection intensity ranged from 2 × 10^3^ to 8 × 10^4^ OPG with maximum shedding at 14 dpi. In 40-day-old chickens, oocysts were first detected at 9 dpi. The infection intensity ranged from 1 × 10^3^ to 3.5 × 10^4^ OPG with maximum shedding at 11 dpi. Likewise, oocysts of *C. avium* were microscopically detected at 9 dpi in ducks, peaking twice at 12 and 17 dpi. The infection intensity ranged from 1 × 10^3^ to 8 × 10^4^ OPG with maximum shedding at 17 dpi. The patterns of oocyst shedding in chickens and ducks are presented in Fig. 2.

### Molecular characterization

All isolates of *C. avium* (from naturally infected cockatiels, one-day-old Roman chickens, three-day-old Roman chickens, three-day-old ducks and 40-day-old Roman chickens) were positive for the *SSU* rRNA gene, actin gene and *HSP70* gene by PCR, but the *COWP* gene was not present. The *SSU* rRNA gene, actin gene and HSP70 gene nucleotide sequence obtained in this study shared 100% identity with *Cryptosporidium avian* genotype V obtained from AY271721, AB471661 and AB538401, respectively.

The PCR-RFLP analysis indicated that the *SSU* rRNA gene of *C. avium* was cut into two pieces by *Ssp*I endonuclease, 497 bp and 253 bp, respectively (see Additional file 1: Figure S1a). By using the *VspI* endonuclease, the *SSU* rRNA gene of *C. avium* was cut into three pieces, 621 bp, 115 bp and 104 bp, respectively (see Additional file 1: Figure S1b). The sequences have been deposited in the GenBank database under accession numbers JQ246415 (*SSU* rRNA gene), JQ320301 (actin gene) and JQ798893 (*HSP70* gene).

### Histological and ultrastructural observation

**Fig. 3:**
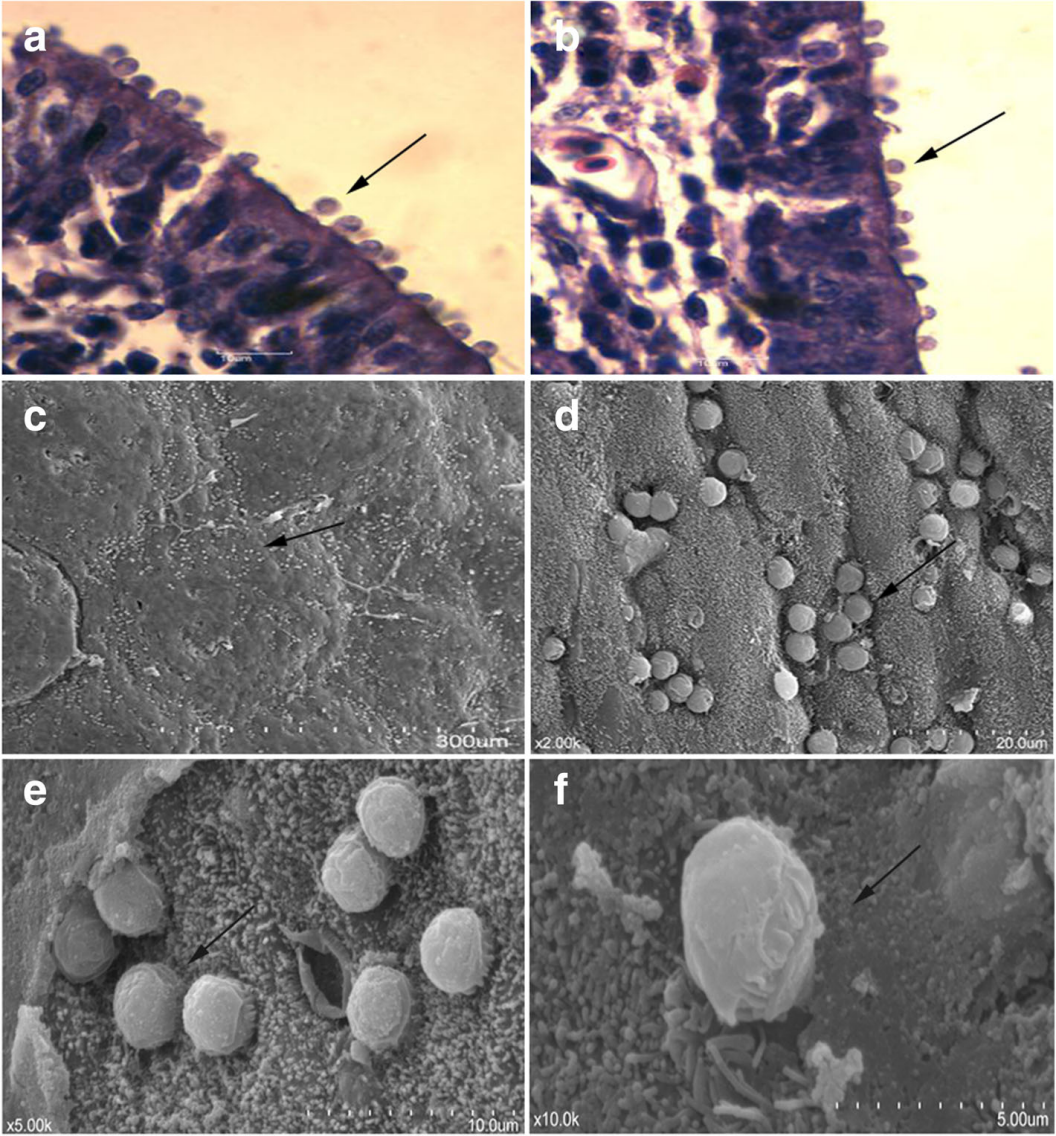
Histological observation of *C. avium* infection in hens (15 dpi) using H&E staining and scanning electronic microscopic observation. *Cryptosporidium* infection was found in the epithelial cells of the bursa fabricii (BF) (**a**, **b**, ×1000) (arrows). *Cryptosporidium* infection was more pronounced in the longitudinal plica of the BF when histologically observed by scanning electron microscopy (**c**-**f**, arrows)

The results of tissue section showed that *Cryptosporidium* infection in chickens (at 15 dpi) was only found in the epithelial cells of the BF (Fig. 3a, b). A large number of developmental stages of *C. avium* had adhered to the surface of the BF. This phenomenon was more pronounced in the longitudinal plica of the BF, when histologically observed by scanning electron microscopy. The BF was almost completely covered with *C. avium* at different endogenous stages (Fig. 3c-f). However, no *Cryptosporidium* developmental stages or pathological changes were observed in other host organs, including the liver, trachea, stomach or intestines. No pathological changes were observed.

## Discussion

*Cryptosporidium* species are important zoonotic protozoans that infect a wide range of hosts [29]. More than 37 species of *Cryptosporidium* have been formally described and are considered valid [30]. Although several *Cryptosporidium* genotypes/isolates have been reported in birds, only four species are considered avian-adapted because of a lack of the biological and morphological data necessary for species designation [31, 32]. Here, we revisit the infectivity and pathogenicity of *C. avium* and provide new information on the biology of this species.

Cryptosporidiosis has been reported in more than 30 avian species worldwide, primarily causing respiratory and enteric infections in birds [1, 33]. *Cryptosporidium avium*, which is naturally detected in the red-crowned parakeet (*Cyanoramphus novaezealandiae*), rosy-faced lovebird (*Agapornis roseicollis*), chicken (*Gallus gallus*), blue-fronted Amazon (*Amazona aestiva*), Mitchell’s cockatoo (*Lophochroaleadbeateri*), cockatiel (*Nymphicus holandicus*) and budgerigar (*Melopsittacus undulatus*), can also infect hens (*Gallus gallus domesticus*) and budgerigars (*Melopsittacus undulatus*) in experimental models [1, 5, 12, 34–37]. In the present study, fecal examination of infected animals revealed fully sporulated *C. avium* oocysts in hens and ducks. No C*ryptosporidium* oocysts were found in quail, BALB/c mice or Mongolian gerbils during the experiment. This finding is consistent with a previously study [5]. However, C*ryptosporidium* oocysts were not found in 20-day-old budgerigars; this difference may be influenced by the age of the experimental budgerigars.

In the present study, we have shown that the prepatent period of *C. avium* was eight and nine days in hens and ducks, respectively, similar to a previous study [5]. In chickens infected with *C. baileyi*, the prepatent period was three dpi [3]. In two nine-day-old chickens, oocysts were excreted for six consecutive days, beginning 25 days after feeding on *C. galli* oocysts [4]. The variability in the prepatent and patent period may depend on the species of *Cryptosporidium* and the status of experimental animals.

Differences in pathogenicity between *Cryptosporidium* species and genotypes have been reported in birds [38–40]. *Cryptosporidium baileyi* and avian genotype II are generally regarded as etiological agents for infections in the ocular conjunctiva, respiratory tract, BF, rectum and cloaca [41]. In domestic chickens and turkeys, infection with *C. meleagridis* causes subclinical infection or clinical signs related to intestinal infection [42, 43]. Infection with *C. galli*, *C. muris*, and avian genotype III are characterized by chronic gastric disease, with clinical signs that include vomiting, weight loss, and macroscopic and microscopic lesions in the proventriculus [44, 45]. In birds infected with *C. avium*, oocysts were detected in the kidney, ureter and cloaca in natural infections, and the ileum and cecum following experimental infection [5, 37]. In this study, however, developmental stages of *C. avium* were mainly observed in longitudinal plica of the BF, with high numbers in histological sections and SEM study. This finding indicates that the BF may be the main parasitic site of *C. avium* infection.

## Conclusions

We have revisited the infectivity and pathogenicity of *C. avium* in several species of animals. Compared to previous studies, oocysts of *C. avium* were mainly detected in the BF of three-day-old hens at 15 dpi. This reveals that the BF may be the main site of *C. avium* infection. All findings in the present study provide new information on the biology of *C. avium.*

## Additional file

Additional file 1: **Figure S1. a** PCR-RFLP products with *Ssp*I restriction enzyme. Two cuttings in locations 497 and 253 bp are visible on gel electrophoresis. **b** PCR-RFLP products with *Vsp*I restriction enzyme. Three cuttings in locations 104, 115, and 621 bp are visible on agarose gel. Lane M: DNA size marker; Lanes 1–5: positive *Cryptosporidium* samples (naturally infected oocysts, passaged oocysts, oocysts in 3-day-old hen, oocysts in 40-day-old hen, oocysts in 3-day-old duck, respectively); Lane P: positive control for *Cryptosporidium*; Lane N: negtive control (molecular grade water). (TIF 1765 kb)

## Abbreviations

BF: Bursa fabricii
dpi: Days post-infection
