# Supplementary figures and images for: Revisiting the infectivity and pathogenicity of Cryptosporidium avium provides new information on parasitic sites within the host
Source: Parasit Vectors. 2018 Sep 19;11:514. doi: 10.1186/s13071-018-3088-x (PMC6146613; doi:10.1186/s13071-018-3088-x)

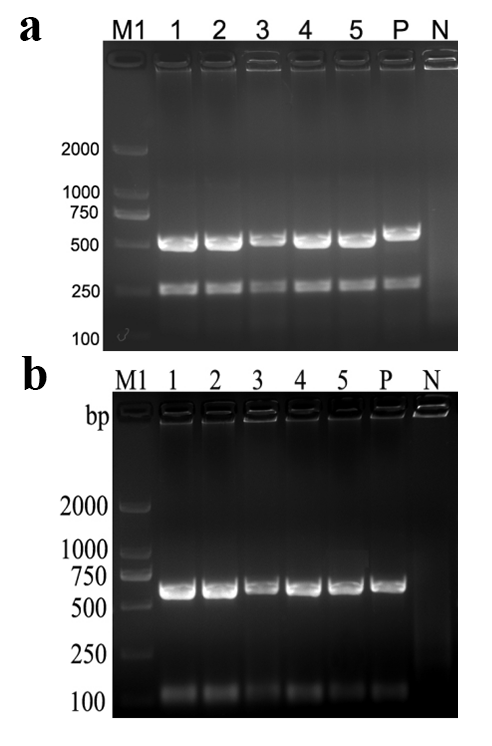

Supplement: Supplementary file 1 — Figure S1. a PCR-RFLP products with SspI restriction enzyme. Two cuttings in locations 497 and 253 bp are visible on gel electrophoresis. b PCR-RFLP products with VspI restriction enzyme. Three cuttings in locations 104, 115, and 621 bp are visible on agarose gel. Lane M: DNA size marker; Lanes 1–5: positive Cryptosporidium samples (naturally infected oocysts, passaged oocysts, oocysts in 3-day-old hen, oocysts in 40-day-old hen, oocysts in 3-day-old duck, respectively); Lane P: positive control for Cryptosporidium; Lane N: negtive control (molecular grade water). (TIF 1765 kb) [file 13071_2018_3088_MOESM1_ESM.tif]
